# Fight or flight mechanism and sports activities: psychophysiological aspects

**DOI:** 10.1192/j.eurpsy.2023.1294

**Published:** 2023-07-19

**Authors:** V. Romaniuk, S. Fedorchuk

**Affiliations:** 1 Rivne State Humanitarian University, Rivne; 2 National University of Ukraine on Physical Education and Sport, Kyiv, Ukraine

## Abstract

**Introduction:**

Specialists in biology, medicine, and psychology need to pay special attention to theoretical and practical research on the implementation of the fight or flight mechanism in humans in conditions of war and hostilities. Adaptation mechanism of fight or flight is related to individual and social forms of behavior among people and animals, and is aimed at the homeostasis preservation in difficult living conditions. The evolutionary fight or flight mechanism in the functional connection with stress as a general adaptation syndrome and stress reactivity has individual features due to the strengthening of the functions of certain body systems. The systemic mechanism of fight or flight is accompanied by the corresponding functional and clinical symptoms and significantly influences physical and mental health of a person in various conditions of activity.

**Objectives:**

In this regard, the main goal of this work was to determine the relationship between the behavioral strategy of fighting or escaping under stress and during sports activities.

**Methods:**

The following research methods were used to realize the goal of the work: theoretical analysis and generalization of literary sources and Internet data.

**Results:**

In our opinion, possible versions of Walter Cannon’s concept of the fight-or-flight mechanism are: 1) attack-or-flight mechanism; 2) defense or escape mechanism; 3) pursuit or escape mechanism. At the same time, it is advisable to consider aggression as a struggle, and escape as a struggle. In addition, in line with the concept of Walter Cannon and Hans Selye, it is possible to distinguish the stress of struggle and the stress of flight. Any intense muscle activity is accompanied by changes in the hormonal and nervous regulation of metabolism, as a result of which the body adapts to a certain physical load. In the athlete, these functional changes are observed even before the start of physical exercises, in particular, in the conditions of the pre-start and start state. The nature and reaction of neuro-endocrine mechanisms in the pre-start state depend on the nature of the load, as well as on the individual characteristics of the athlete (age, gender, type of nervous system, temperament, character, training, sports experience, etc.). It is important that the pre-start changes in metabolism contribute to the mobilization of the athlete’s functional potential even before the start of the corresponding physical activity. Moderate activation of the neuro-endocrine mechanisms of the athlete’s body (“combat readiness” state) is optimal. Functionally, it is less beneficial for the athlete’s body to have a sharp strengthening of neuro-endocrine mechanisms (“pre-start fever” state) or their paradoxical inhibition (“pre-start apathy” state).

**Conclusions:**

Thus, in humans, sports activities include a behavioral mechanism of fight or flight in combination with physiological and psychological stress.

**Disclosure of Interest:**

None Declared

